# Precore/Core Region Mutations in Hepatitis B Virus DNA Predict Postoperative Survival in Hepatocellular Carcinoma

**DOI:** 10.1371/journal.pone.0133393

**Published:** 2015-07-24

**Authors:** Ying Xie, Shufeng Liu, Yufei Zhao, Lan Zhang, Yue Zhao, Binghui Liu, Zhanjun Guo

**Affiliations:** 1 Hebei Key Lab of Laboratory Animal Science, Hebei Medical University, Shijiazhuang, Hebei Province, P.R. China; 2 Department of Gastroenterology and Hepatology, The Fourth Hospital of Hebei Medical University, Shijiazhuang, P.R. China; 3 Institute of Laboratory Animal Science, Peking Union Medical College, Peking, P.R. China; CRCL-INSERM, FRANCE

## Abstract

Hepatitis B virus (HBV) DNA is prone to mutations because of the proofreading deficiencies of HBV polymerase. We have identified hepatocellular carcinoma (HCC) survival-associated HBV mutations in the X protein region of HBV DNA. In the present study, we extend our research to assess HCC survival-associated HBV mutations in the HBV precore/core (PreC/C) region. The PreC/C region was amplified and sequenced and the HBV mutations were identified according to the NCBI database (http://www.ncbi.nlm.nih.gov/genome/5536). The relationships between the mutations in the PreC/C region and HCC survival were analyzed. Survival curves were generated using the Kaplan-Meier method, and comparisons between the curves were made using the log-rank test. Multivariate survival analysis was performed using a Cox proportional hazards model. After adjusting for clinical characteristics, the 1915, 2134, 2221, 2245 and 2288 mutational sites were identified as statistically significant independent predictors of HCC survival by multivariate survival analysis using a Cox proportional hazards model. In addition, the mutational site of 1896 was identified for its association with survival at a borderline significance level. A total of five mutations in the precore/core region were identified as independent predictors of postoperative survival in HCC patients. The analysis of HBV DNA mutations may help identify patient subgroups with poor prognosis and may help refine therapeutic decisions regarding HCC patients.

## Introduction

Hepatocellular carcinoma is the third leading cause of cancer deaths globally, resulting in over half a million deaths every year [1]. Despite improved clinical detection methods and therapies, the prognosis of postoperative HCC patients is still poor due to a high recurrence rate. Until now, the molecular mechanism of HCC carcinogenesis has not been fully understood. There could be a vast number of prognostic factors and predictors of the disease, including tumor size, tumor quantity, cell differentiation, venous invasion, and degree of inflammation [2–5]. It has been reported that in Asia and Africa, where hepatitis B virus (HBV) and hepatitis C virus (HCV) are prevalent, the incidence of HCC increased dramatically in recent decades. Notably, almost 80% of HCC cases worldwide were triggered by infection with HBV. Thus, research on HBV has become a hotspot and drawn the attention of clinicians in the field of HCC research.

Hepatitis B virus (HBV) is a hepatotropic virus that chronically infects approximately 300 million people worldwide and is thought to be responsible for a million deaths annually (http://www.ncbi.nlm.nih.gov/genome/5536). Eight HBV genotypes (genotypes A to H) have been identified with a distinct geographic distribution. Among them, genotypes B and C are the predominant HBV genotypes in Eastern Asia. In China, HBV infection is a challenging health issue. Approximately 93 million people are HBV carriers, and 30 million are suffering from chronic hepatitis B [6,7].

The HBV genome contains four overlapping open reading frames that encode the surface protein (S), the core protein, a polymerase, and a multifunctional nonstructural protein called X. HBV DNA is prone to mutations due to the proofreading deficiencies of HBV polymerase [8,9]. The cancer risk of HBV-related HCC (HBV-HCC) and HBV mutations have been well studied in recent years [10–15], but only a few studies have focused on the prognostic value of these mutations in HBV-HCC patients. By assessing HBV DNA in liver tissue specimens, Yeh et al. showed that the basal core promoter A1762T/G1764A mutation is independently predictive of postoperative survival in HCC patients [16]. In addition, our previous study identified HBV-HCC survival-associated HBV mutations in the X protein region [17]. In this paper, our research was extended to assess HCC survival-associated HBV mutations in HBV precore/core (PreC/C) regions.

## Materials and Methods

### Tissue specimens and DNA extraction

HCC tissue specimens were collected at the Fourth Hospital of Hebei University from patients who underwent tumor resection in the Department of Hepatobiliary Surgery between 2007 and 2009. All of the patients were proven to be infected with HBV and lacking other diseases (i.e., HCV, autoimmune hepatitis). Genomic DNA was extracted immediately with a Wizard Genomic DNA extraction kit (Promega, Madison). All procedures were supervised and approved by the Human Tissue Research Committee of the Fourth Hospital of Hebei Medical University, and written inform consent were obtained from each patients.

### Virological Assay

The HBV DNA concentration was quantified using the ABI 7300 TaqMan platform (Life Technologies, Carlsbad, CA) by real-time PCR, and its levels in HCC tissue were calculated as copies per microgram of genomic DNA. HBV genotypes were determined by the multiplex-PCR as described previously [18]. The DNA sequences flanking the PreC/C regions were amplified with the primer pairs listed in Table 1 according to the NCBI database (http://www.ncbi.nlm.nih.gov/genome/5536). Cyclic sequencing was performed with a BigDye Terminator v3.1 Cycle Sequencing Kit (Life Technologies), and the products were separated using an ABI PRISM 3100 Genetic Analyzer (Life Technologies). Mutations were confirmed by repeating the analysis on both strands.

**Table 1:** Primer pairs used in amplifying and sequencing the precore/core regions.

Primer	Nucleotide sequence (5’→3’)	Position	
**HBV1F**	CACCTCTGCACGTAGCAT	1592–1609	Sense
**HBV1R**	TCCCGATACAGAGCAGAG	2002–2019	Antisense
**HBV2F**	TGCCTTCTGACTTCTTTCC	1956–1974	Sense
**HBV2R**	TGAGATTCCCGAGATTGA	2428–2445	Anti-sense

### Statistical analysis

Survival curves were generated using the Kaplan-Meier method, and comparisons between the curves were made using the log-rank test. Multivariate survival analysis was performed using a Cox proportional hazards model. The χ^2^ test was used to analyze dichotomous values among genotype, HBV DNA copy number, mutation number and E antigen status. All of the statistical analyses were performed using the SPSS 18.0 software package (IBM Corporation, Armonk, NY), and a *p* value < 0.05 was considered statistically significant.

## Results

### Clinical characteristics of HBV-HCC patients

A total of 119 HBV-HCC patients were enrolled in this study; eight patients were lost during follow-up, which was performed every three months for 3 years, and the remaining 111 patients were genotyped as follows: two were A, two were D, 58 were B, 46 were C, one was B+C, one was C+D and one was A+C. As the number of patients in groups A and D is too samll, we used 104 patients of B and C types to further analysis. The relationships between the clinical characteristics and postoperative overall survival in HBV-HCC patients were assessed using the Kaplan—Meier method and the log-rank test. The characteristics of tumor size, tumor stage, and portal vein thrombosis were associated with survival in these patients (Table 2). Based on multivariate analysis with the Cox proportional hazards model, tumor size and portal vein thrombosis were identified as independent predictors of postoperative survival in HBV-HCC patients.

**Table 2:** Univariate and multivariate analysis of clinical factors associated with postoperational survival in HBV-HCC patients.

Factors	No. of	3-year	Univariate	Multivariate analysis		
	case	survival rate	analysis *p*-value	*p*-value	RR^a^	95%CI
**Age**			0.559	0.483	0.828	0.488–1.404
** ≤55**	54	44.8%				
** >55**	50	39.1%				
**Sex**			0.547	0.343	1.537	0.632–3.738
** male**	92	41.3%				
** female**	12	50.0%				
**CHILD classification**			0.160	0.294	1.631	0.653–4.072
** A**	96	43.8%				
** B**	8	25.0%				
**Genotype**			0.559	0.176	1.461	0.844–2.531
** B**	58	44.8%				
** C**	46	39.1%				
**Tumor quantity**			0.611	0.122	1.755	0.860–3.580
** single**	87	42.5%				
** multiple**	17	41.2%				
**Portal vein thrombosis**			<0.001	0.023	2.276	1.120–4.625
** Yes**	14	7.1%				
** No**	90	47.8%				
**TNM classification**			0.008	0.091	1.688	0.920–3.096
** I**	48	58.3%				
** II+ III**	56	28.6%				
**Tumor size (diameter)**			0.007	0.010	2.134	1.202–3.786
** ≤5cm**	43	55.8%				
** >5cm**	61	32.8%				
**HBV DNA** ^**b**^			0.033	0.034	0.550	0.316–0.957
** <6.0E+08**	53	34.0%				
** ≥6.0E+08**	51	51.0%				

^a^RR: Relative Risk;

^b^Defined as copies per microgram of genomic DNA.

### Virological parameters associated with postoperative survival in HCC patients

**Fig 1:**
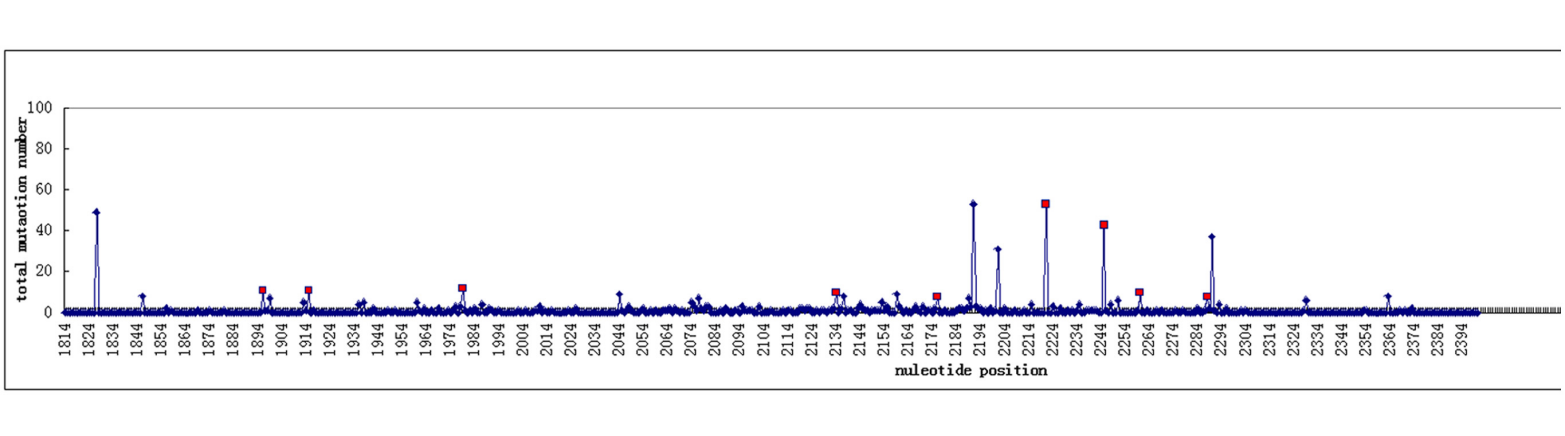
Distribution of mutation frequency in the PreC/C region. The square indicates the mutations associated with HBV-HCC survival by univariate analysis using the log-rank test, and the rhombus indicates mutations that were not associated with HBV-HCC.

The DNA sequences flanking the PreC/C region were sequenced, and a total of 186 mutations were identified in the PreC/C region. The highest mutation rate was observed at the 2221 nucleotide with 55 carriers (Fig 1).

The relationship between HBV mutations and HBV-HCC survival was analyzed with the log-rank test and Cox proportional hazards model, and 19 mutational sites with mutational rates higher than 5% in HCC patients were used for survival analysis, as shown in Tables 3 and 4. Among these 19 sites, the following nine sites were associated with postoperative survival at statistically significant levels in HCC tissue based on the log-rank test with the Kaplan-Meier method: nucleotides 1896, 1915, 1979, 2134, 2176, 2221, 2245, 2260, and 2288. Almost half of the detected mutations (9/19) were associated with overall survival in HCC (Fig 1), and more than a half of them (5/9) were associated with worse outcome by multivariate analysis with the Cox proportional hazard model (Tables 3 and 4). As shown in Tables 3 and 4, after adjusting for clinical characteristics, the following five mutational sites were identified as independent predictors of HCC survival at statistically significant levels: 1915 (RR:0.175, 95%CI: 0.040–0.755, *p* = 0.020), 2134 (RR: 3.803,95%CI: 1.772–8.162, *p* = 0.001), 2221 (RR: 2.490, 95%CI: 1.433–4.325, *p* = 0.001), 2245 (RR: 2.056, 95%CI:1.215–3.482, *p* = 0.007) and 2288 (RR: 2.594, 95%CI: 1.144–5.879, *p* = 0.022). Additionally, one mutational site was associated with survival at a borderline significance level: 1896 (RR: 2.044, 95%CI: 0.982–4.257, *p* = 0.056). We associated the mutations and clinial factors, and no association has been foound (data not shown).

**Table 3:** Univariate and multivariate analysis of precore/core mutations associated with postoperative survival in patients with HBV-HCC (nucleotide 1814–2160).

Nucleotide site		3-year	Univariate	Multivariate analysis				
	No. of case	survival rate	analysis *p*-value	*p*-value	RR	95%CI	Amino acid substitution	
**1846**			0.817	0.604	0.777	0.300–2.015	No	
**A**	95	42.1%						
**T**	9	44.4%						
**1889**			0.720	0.869	0.924	0.359–2.378	Yes	Trp→Arg(26)
**T**	96	42.7%						
**A**	8	37.5%						
**1896**			0.028	0.056	2.044	0.982–4.257	Yes	Trp→stop(28)
**G**	93	45.2%						
**A**	11	18.2%						
**1915**			0.015	0.020	0.175	0.040–0.755	No	
**G**	93	38.0%						
**A or C**	11	81.8%						
**1979**			0.050	0.505	1.294	0.606–2.764	Yes	Ile→Val(56)
**A**	92	45.7%						
**G**	12	16.7%						
**2044**			0.471	0.582	0.765	0.295–1.983	No	
**T**	94	41.5%						
**C**	10	50.0%						
**2077**			0.429	0.625	0.743	0.226–2.441	No	
**T**	97	41.2%						
**A or C**	7	57.1%						
**2134**			0.000	0.001	3.803	1.772–8.162	No	
**C**	94	45.7%						
**T**	10	10.0%						
**2137**			0.736	0.551	1.382	0.477–4.002	No	
**A**	96	41.7%						
**G or T or C**	8	50.0%						
**2159**			0.759	0.570	1.314	0.512–3.373	Yes	Ser→Arg(116)
**C**	96	42.7%						
**G**	8	37.5%						

Abbreviations: ORF, open reading frame; PreC/C, precore/core

**Table 4:** Univariate and multivariate analysis of precore/core mutations associated with postoperative survival in patients with HBV-HCC (nucleotide 2161–2452).

Nucleotide site		3-year	Univariate	Multivariate analysis				
	No. of case	survival rate	analysis *p*-value	*p*-value	RR	95%CI	Amino acid substitution	
**2176**			0.012	0.139	1.829	0.823–4.065	No	
**T**	95	45.3%						
**C**	9	11.1%						
**2189**			0.782	0.926	0.944	0.277–3.212	Yes	Ile→Phe(126)
**A**	98	41.8%						
**T**	6	50.0%						
**2201**			0.580	0.364	1.288	0.745–2.227	No	
**T**	72	44.4%						
**C**	32	37.5%						
**2221**			0.001	0.001	2.490	1.433–4.325	No	
**C**	49	59.2%						
**T**	55	27.3%						
**2245**			0.050	0.007	2.056	1.215–3.482	No	
**C**	60	50.0%						
**T**	44	31.8%						
**2251**			0.674	0.342	0.554	0.164–1.876	No	
**G**	98	41.8%						
**A**	6	50.0%						
**2260**			0.029	0.406	1.379	0.646–2.944	No	
**G**	93	45.2%						
**A**	11	18.2%						
**2288**			0.002	0.022	2.594	1.144–5.879	Yes	Pro→Ser(159)
**C**	96	45.8%						
**A**	8	0.0%						
**2290**			0.202	0.266	1.359	0.791–2.333	No	
**C**	67	46.0%						
**T**	37	35.1%						

Abbreviations: ORF, open reading frame; PreC/C, precore/core

## Discussion

We identified the HBV-HCC survival-associated HBV mutations in the X protein region in a previous study [17]. In this study, we extended our research to evaluate the mutations in the HBV DNA sequences flanking PreC/C regions and identified six mutations associated with HBV-HCC survival by multivariate analysis. Our results demonstrate that the HBV mutations are not only involved in hepatocarcinogenesis, as reported previously, but also affect postoperative prognosis.

HBV infection remains the major health challenge in Southeast Asia, where HBV is highly prevalent [16]. The precore/core (PC/C) is one of the four HBV open reading frames (ORFs), which encodes for both the hepatitis B e antigen (HBeAg) and the core protein (HBcAg). HBeAg expression may be reduced by the precore mutations G1896A and the double mutation A1762T/G1764A in the basal core promoter (BCP) region [19]. In the past decade, the two mutations were identified that were contributed to the clinical severity of HCC [20–22]. Six HCC survival associated mutations, 1896, 1915, 2134, 2221, 2245, and 2288, have been identified in the PreC/C region in our work. Among them, G1896A and C2288A can introduce mutations in amino acid residues. However, only G1896A has been well studied. The G1896A mutation prevents the production of HBeA by introducing a stop codon into the open reading frame of the precore region and was shown to be associated with hepatocarcinogenesis [8,22]. Kim et al. verified that several preC/C mutations that can introduce codon mutation are linked with the HCC [15]. The results from this study indicated that the mutations other than G1896A in the precore/core region were associated with the HCC outcome regardless of whether it causes codon mutation. Validation in both other populations and laboratory-based functional studies is still required to elucidate the way in which these mutations affected the HCC progress and the difference between the two mutation types in introducing codon mutations. We performed multivariate analysis including the HCC survival associated clinical factors and these five HCC survival mutations to exclude the interactions among the mutations, the 1915 (RR:0.208, 95%CI: 0.050–0.867, *p* = 0.031) and 2134 (RR:2.644, 95%CI: 1.201–5.819, *p* = 0.016) mutations display the survival association at significantly statistical level, the 2221 (RR:2.085, 95%CI: 0.990–4.392, *p* = 0.053) mutation display association at borderline levels. The analysis of HBV DNA mutations may help identify patient subgroups with poor prognoses and may help refine therapeutic decisions regarding HCC patients.

## References

[1] Gomaa AI, Khan SA, Toledano MB, Waked I, Taylor-Robinson SD. Hepatocellular carcinoma: epidemiology, risk factors and pathogenesis. World J Gastroenterol. 2008;14(27):4300–8. 18666317 10.3748/wjg.14.4300 PMC2731180

[2] Maki A, Kono H, Gupta M, Asakawa M, Suzuki T, et al Predictive power of biomarkers of oxidative stress and inflammation in patients with hepatitis C virus-associated hepatocellular carcinoma. Ann Surg Oncol. 2007;14(3):1182–90. 17195915 10.1245/s10434-006-9049-1

[3] Okada S, Shimada K, Yamamoto J, Takayama T, Kosuge T, et al Predictive factors for postoperative recurrence of hepatocellular carcinoma. Gastroenterology. 1994;106 (6):1618–24. 8194710 10.1016/0016-5085(94)90419-7

[4] Minagawa M, Makuuchi M, Takayama T, Kokudo N. Selection criteria for repeat hepatectomy in patients with recurrent hepatocellular carcinoma. Ann Surg. 2003; 238(5):703–10. 14578733 10.1097/01.sla.0000094549.11754.e6 PMC1356149

[5] Wang C, Zhang F, Fan H, Peng L, Zhang R, et al Sequence polymorphisms of mitochondrial D-loop and hepatocellular carcinoma outcome. Biochem Biophys Res Commun. 2011; 406 (3):493–6. Epub 2011/02/ 21. 10.1016/j.bbrc.2011.02.088 21345333

[6] Yu MC, Yuan JM, Govindarajan S, Ross RK. Epidemiology of hepatocellular carcinoma. Can J Gastroenterol. 2000;14(8):703–9. 11185536 10.1155/2000/371801

[7] Lu FM, Zhuang H. Management of hepatitis B in China. Chin Med J (Engl). 2009; 122(1):3–4. 19187608

[8] Yang HI, Yeh SH, Chen PJ, Iloeje UH, Jen CL, et al Associations between hepatitis B virus genotype and mutants and the risk of hepatocellular carcinoma. J Natl Cancer Inst. 2008;100(16):1134–43. Epub 2008/08/11. 10.1093/jnci/djn243 18695135 PMC2518166

[9] Tsai WL, Chung RT. Viral hepatocarcinogenesis. Oncogene. 2010;29(16):2309–24. Epub 2010/03/15. 10.1038/onc.2010.36 20228847 PMC3148694

[10] Chan HL, Tse CH, Mo F, Koh J, Wong VW, et al High viral load and hepatitis B virus subgenotype ce are associated with increased risk of hepatocellular carcinoma. J Clin Oncol. 2008; 26(2):177–82. 18182659 10.1200/JCO.2007.13.2043

[11] Yu MW, Yeh SH, Chen PJ, Liaw YF, Lin CL, et al Hepatitis B virus genotype and DNA level and hepatocellular carcinoma: a prospective study in men. J Natl Cancer Inst. 2005;97(4):265–72. 15713961 10.1093/jnci/dji043

[12] Liu CJ, Chen BF, Chen PJ, Lai MY, Huang WL, et al Role of hepatitis B viral load and basal core promoter mutation in hepatocellular carcinoma in hepatitis B carriers. J Infect Dis. 2006; 193(9):1258–65. Epub 2006/04/04. 16586363 10.1086/502978

[13] Lin CL, Liu CH, Chen W, Huang WL, Chen PJ, et al Association of pre-S deletion mutant of hepatitis B virus with risk of hepatocellular carcinoma. J Gastroenterol Hepatol. 2007;22(7):1098–103. 17608857 10.1111/j.1440-1746.2006.04515.x

[14] Kao JH, Chen PJ, Lai MY, Chen DS. Basal core promoter mutations of hepatitis B virus increase the risk of hepatocellular carcinoma in hepatitis B carriers. Gastroenterology. 2003; 124 (2):327–34. 12557138 10.1053/gast.2003.50053

[15] Kim DW, Lee SA, Hwang ES, Kook YH, Kim BJ. () Naturally occurring precore/core region mutations of hepatitis B virus genotype C related to hepatocellular carcinoma. PLOS ONE. 2012;7(10):e47372 Epub 2012 /10/10. 10.1371/journal.pone.0047372 23071796 PMC3468518

[16] Yeh CT, So M, Ng J, Yang HW, Chang ML, et al Hepatitis B virus-DNA level and basal core promoter A1762T/G1764A mutation in liver tissue independently predict postoperative survival in hepatocellular carcinoma. Hepatology. 2010;52 (6):1922–33. Epub 2010 /09/02. 10.1002/hep.23898 20814897

[17] Xie Y, Liu S, Zhao Y, Guo Z, Xu J. X protein mutations in hepatitis B virus DNA predict postoperative survival in hepatocellular carcinoma. Tumour Biol. 2014;35(10):10325–31. Epub 2014/07/19. 25034530 10.1007/s13277-014-2331-0

[18] Chen J, Yin J, Tan X, Zhang H, Zhang H, et al Improved multiplex-PCR to identify hepatitis B virus genotypes A-F and subgenotypes B1, B2, C1 and C2. J Clin Virol. 2007; 38(3):238–43. 17224304 10.1016/j.jcv.2006.12.007

[19] Yousif M, Bell TG, Mudawi H, Glebe D, Kramvis A. Analysis of ultra-deep pyrosequencing and cloning based sequencing of the basic Core promoter/precore/core region of hepatitis B virus using newly developed bioinformaticstools. PLOS ONE. 2014;9(4):e95377 10.1371/journal.pone.0095377 eCollection 2014. 24740330 PMC3989311

[20] Liu CJ, Chen BF, Chen PJ, Lai MY, Huang WL, et al Role of hepatitis B virus precore/core promoter mutations and serum viral loadon noncirrhotic hepatocellular carcinoma: a case-control study. J Infect Dis. 2006;194(5):594–9. Epub 2006 /07/18. 16897657 10.1086/505883

[21] Zheng JX, Zeng Z, Zheng YY, Yin SJ, Zhang DY, et al Role of hepatitis B virus base core and precore/core promoter mutations on hepatocellularcarcinoma in untreated older genotype C Chinese patients. 2011;18(10):e423–31. Epub 2011 /04/4. 10.1111/j.1365-2893.2011.01458.x 21914059

[22] Jang JW, Chun JY, Park YM, Shin SK, Yoo W, et al Mutational complex genotype of the hepatitis B virus X /precore regions as a novel predictive marker for hepatocellular carcinoma. Cancer Sci.2012; 103:296–304. Epub 2012/01/ 19. 10.1111/j.1349-7006.2011.02170.x 22136288

